# Minocycline Attenuates Microglia/Macrophage Phagocytic Activity and Inhibits SAH-Induced Neuronal Cell Death and Inflammation

**DOI:** 10.1007/s12028-022-01511-5

**Published:** 2022-05-18

**Authors:** Kinga G. Blecharz-Lang, Victor Patsouris, Melina Nieminen-Kelhä, Stefanie Seiffert, Ulf C. Schneider, Peter Vajkoczy

**Affiliations:** 1 Institute of Experimental Neurosurgery, Charité – Universitätsmedizin Berlin, Charitéplatz 1, 10117 Berlin, Germany; 2 Department of Neuropathology, Charité – Universitätsmedizin Berlin, Berlin, Germany; 3 Department of Neurosurgery, Charité – Universitätsmedizin Berlin, Berlin, Germany; 4 Center for Stroke Research Berlin, Charité – Universitätsmedizin Berlin, Berlin, Germany

**Keywords:** Anti-inflammatory agents, Apoptosis, Inflammation, Microglia, Cytokines, Phagocytosis, Subarachnoid hemorrhage

## Abstract

**Background:**

Neuroprotective treatment strategies aiming at interfering with either inflammation or cell death indicate the importance of these mechanisms in the development of brain injury after subarachnoid hemorrhage (SAH). This study was undertaken to evaluate the influence of minocycline on microglia/macrophage cell activity and its neuroprotective and anti-inflammatory impact 14 days after aneurismal SAH in mice.

**Methods:**

Endovascular filament perforation was used to induce SAH in mice. SAH + vehicle-operated mice were used as controls for SAH vehicle-treated mice and SAH + minocycline-treated mice. The drug administration started 4 h after SAH induction and was daily repeated until day 7 post SAH and continued until day 14 every second day. Brain cryosections were immunolabeled for Iba1 to detect microglia/macrophages and NeuN to visualize neurons. Phagocytosis assay was performed to determine the microglia/macrophage activity status. Apoptotic cells were stained using terminal deoxyuridine triphosphate nick end labeling. Real-time quantitative polymerase chain reaction was used to estimate cytokine gene expression.

**Results:**

We observed a significantly reduced phagocytic activity of microglia/macrophages accompanied by a lowered spatial interaction with neurons and reduced neuronal apoptosis achieved by minocycline administration after SAH. Moreover, the SAH-induced overexpression of pro-inflammatory cytokines and neuronal cell death was markedly attenuated by the compound.

**Conclusions:**

Minocycline treatment may be implicated as a therapeutic approach with long-term benefits in the management of secondary brain injury after SAH in a clinically relevant time window.

**Supplementary Information:**

The online version contains supplementary material available at 10.1007/s12028-022-01511-5.

## Introduction

Acute subarachnoid hemorrhage (SAH), caused by the rupture of an intracranial aneurysm, contributes for a third of all hemorrhagic strokes. However, unlike all other ischemic or hemorrhagic strokes, SAH is a strictly extraparenchymal disease occuring within the basal cisterns. Following the onset of the bleeding, primary brain injury results from an abrupt increase of the intracranial pressure and a consecutive decrease of the cerebral perfusion. The subarachnoid blood then directly activates an inflammatory cascade, causing central nervous system (CNS) injury, blood–brain barrier breakdown and culminating by the development of brain edema. Secondary brain injury after SAH has been mainly attributed to ischemic events caused by delayed cerebral vasospasm. However, despite showing a significant prevention/reversal of cerebral vasoconstriction after SAH, large multicenter clinical trials (CONSCIOUS-1–3) failed to demonstrate benefits in clinical outcome [1, 2]. These findings led to the idea that vasospasm may not be the sole mechanism explaining secondary brain injury, rather suggesting that other potential mechanisms that occur within minutes after the bleeding may affect the outcome [3–7]. Recently, we hypothesized that an intraparenchymal activation of the immune system might contribute to secondary brain injury after SAH [8]. We have demonstrated a substantial accumulation of microglia/macrophages with a prominent elevation of inflammatory cytokines secreted by those reaching its maximum extent by 14 days after the bleeding both in human and animal brain samples [8]. Moreover, this increase in the amount of microglia rather than macrophages has been directly associated with the extent of brain damage after SAH due to a significantly reduced neuronal apoptosis in mice depleted of CD11b-positive microglia/macrophages by intraventricular ganciclovir injection [8, 9]. Importantly, intracerebral cytokine concentrations were predictive for the development of secondary brain injury, delayed ischemic events, and brain swelling [10]. Also, an upregulation of inflammatory cytokines in the cerebrospinal fluid of patients suffering from SAH has been identified by our group [11, 12]. As a sign of an intravascular inflammatory response, the activation of leukocytes and thrombocytes seems to modulate the extent of the neuroaxonal damage after the hemorrhage [13, 14].

Beside its antibiotic properties, the semisynthetic broad-spectrum tetracycline minocycline hydrochloride, hereafter referred to as minocycline, has been reported as a promising anti-inflammatory, antiapoptotic, and neuroprotective compound with iron-chelating and antioxidative properties in numerous clinical studies [15, 16]. Undoubtedly, its multifaceted beneficial activities are achieved through conserved primary mechanisms, such as modulation of the p38 mitogen-activated protein kinase and phosphoinositide 3-kinase/Akt signaling pathways and inhibition of matrix metalloproteinases (MMPs), or through its direct inhibition of calcium influx and iron overload [17–19]. The drug has also been reported to target a broad range of secondary injury mechanisms (such as neuronal damage by protecting neurons from oxidative stress), scavenge free radicals, and inhibit inducible nitric oxide synthase (iNOS), glutamate-induced apoptosis, or cytochrome C-induced apoptosis. Minocycline was also shown to counteract activation of microglia/macrophages occurring due to secondary brain injury into an anti-inflammatory phenotype by blocking the p38 mitogen-activated protein kinase pathway [20]. In vivo studies on rodents confirmed this drug to be effective in causing a long-term improvement in functional and cognitive outcome after experimental SAH [21–23]. However, given the multiple effects of this promising, clinically available drug, a detailed investigation determining mechanisms underlying minocycline-mediated neuroprotection resulting from the deactivation of microglia/macrophages in the context of SAH remains outstanding and has to be addressed by further experimental work.

It was therefore the interest in our current study to explore the modulatory effect of minocycline as a straightforward approach to interfere with microglia as the cellular component of the brain’s innate immune system and with macrophages invading the CNS from the periphery on day 14 after SAH. A special attention was paid to the impact of minocycline on the activity of microglia/macrophages on neuronal cell death and on the inflammatory response due to delayed brain injury post SAH.

## Methods

### Animals, Experimental Groups, and SAH Mouse Model

Animal experiments were approved by the responsible ethics committee; conducted according to the regulatory guidelines and standards given by the Landesamt für Gesundheit und Soziales, Berlin; and performed according to the European Convention (ETS 123 of 1986). All efforts were made to minimize suffering and numbers of animals used. All sections in the article were reported according to Animal Research Reporting of In Vivo Experiments guidelines. All diagnostic assessments were done on the basis of our institutional guidelines.

In total, seventy 12- to 14-week-old male C57BL/6 J mice weighing 30–32 g were included in this study. SAH was induced by the endovascular perforation mouse model as described elsewhere [8]. Sham-operated mice underwent the same procedure, except that the suture was immediately withdrawn when the resistance was felt. Standard hematoxylin and eosin staining ruled out intracerebral hemorrhage and territorial infarctions. Animals suffering from hemiparesis following the operation were excluded from experiments. All animals were randomly assigned to one of three groups: sham + vehicle, SAH + vehicle, and SAH + minocycline.

### Minocycline Administration

Minocycline hydrochloride (Sigma-Aldrich) was diluted in phosphate buffered saline (PBS) (0.1 mol/L, pH 7.4; vehicle) and injected intraperitoneally into mice in a final concentration of 45 mg/kg/dose. The drug administration started 4 h after SAH induction and was repeated daily until day 7 post SAH and continued until day 14 every second day. The vehicle group received the same volume of PBS intraperitoneally 4 h after induction of the bleeding.

### Mortality

Animal mortality from all experimental groups, summarized in the Kaplan–Meier curve indicated in Supplementary Fig. S1, was assessed at 1–3 h, 4–6 h, 6–12 h, 12–24 h, and weekly thereafter.

### Acute Brain Slice Preparation

Mice of each experimental group were decapitated on day 14 post SAH. After the skin and skull were opened, the whole brain was removed and washed in ice-cold artificial cerebrospinal fluid (ACSF) (134 mM NaCl, 2.5 mM KCl, 2 mM CaCl_2_, 1.3 mM MgCl_2_, 26 mM NaHCO_3_, 1.25 mM K_2_HPO_4_, 10 mM glucose). Brains were cut transversally in two parts. The forebrain was fixed with glue to a slicing chamber filled with ice-cold ACSF. Horizontal brain slices (300 µm, coronal orientation) were prepared by vibratome VT 1000 S (Leica), placed onto a nylon grid (BD Bioscience), and stored with carbogen (95% CO_2_, 5% O_2_) in ACSF at room temperature for 2 h until phagocytosis assay.

### Phagocytosis Assay

Fluoresbrite YG Carboxylate Microspheres beads (2.00 µm) were coated with heat-inactivated FBS. Slices were transferred into a 24-well plate, and ACSF was exchanged by 500 µl prewarmed equilibrated DMEM (Gibco). Five-microliter beads were added to each well, and the plate was gently shaken. Slices were incubated at 37 °C in 5% CO_2_ for 1 h, washed three times with PBS, subsequently fixed with 4% PFA overnight, and then additionally washed and stained with ionized calcium-binding adaptor molecule-1 (Iba1) antibody.

### Immunohistochemistry and Image Analysis

Immunofluorescence analysis of mouse brain specimens was performed in coronal Sects. (20 µm). Standard hematoxylin and eosin staining and iron staining were performed in all animals to rule out intracerebral hemorrhage and territorial infarctions at day 14. Slides were incubated with primary antibodies at 4 °C overnight. Microglia/macrophages were visualized by staining for Iba1 (rabbit-anti Iba1, WAKO Pure Chemical Industries), and glial fibrillary acidic protein (GFAP) (chicken-anti GFAP, Abcam) and neuronal nuclei (NeuN) (mouse-anti NeuN, Millipore) staining were used to identify reactive astrocytes and neurons, respectively. Secondary antibodies (all obtained from Jackson ImmunoResearch Lab) incubated for 1.5 h at room temperature were as follows: DyLight651 donkey anti-chicken, DyLight651 donkey anti-rabbit, DyLight488 donkey-anti-rabbit, and FITC donkey-anti-mouse. Nuclei were counterstained with 4',6-diamidin-2-phenylindol (DAPI)-containing mounting medium (Dianova).

For quantitative analysis, images were taken by fluorescence microscopy (Zeiss, Axio Observer Z1, Carl Zeiss, Microimaging GmbH) equipped with a digital camera (AxioCam MRc). Image acquisition of confocal microscopy was obtained with a confocal microscope (TCS SP5, Leica) using a z step of 0.1 µm and 63 × 1.4 NA oil immersion objective. Images were acquired using LCF AF software (Leica). Sections were divided into six to ten high-power fields (HPF), allowing for total cell counts per section on three different levels of the brain and bregma 1.5 mm before (approximately the area of thalamus/hypothalamus) and behind (approximately the area of the hippocampus), recalculated per µm^2^. Immunoreactive areas were measured using a computer-assisted image analysis program (ImageJ.net). Images were randomly captured from the same HPF (areas used for examination) and analyzed using the ImageTool software automating the analysis by converting all immune-labeled elements that fell within a threshold range into pure black pixels and the rest of the image into pure white pixels. The software then quantified the total number and percentages of black and white pixels, allowing for statistical analysis. Microglia/macrophage–neuron interaction was specified by the index calculating the number of microglia/macrophages interacting with at least one NeuN-positive cell (interactive microglia/macrophages) per number of all activated microglia/macrophages counted per HPF.

### Neuronal Cell Death

Neuronal cell death was detected by terminal deoxyuridine triphosphate nick end labeling (TUNEL) (ApopTag Red In Situ Apoptosis Detection Kit, Millipore), according to the manufacturer’s protocol, followed by labeling with NeuN and DAPI. Cells triple-positive for TUNEL, NeuN, and DAPI were considered as neurons undergoing cell death.

### Quantitative Real-Time Polymerase Chain Reaction

RNA isolation from whole-brain tissue homogenates (PureLink RNA Mini Kit, Life Technologies), cDNA synthesis (Onestep RT-PCR Kit, Qiagen), and quantitative real-time polymerase chain reaction (qPCR) (Premix ex Taq Perfect Real Time Kit, Takara) were performed in accordance to manufacturers’ instructions, as previously described [14]. All primer sequences used for the qPCR reactions were designed using Primer Express Software and are listed in Table 1. The ABI PRISM 7300 SDS software (relative quantification study) was used to determine the cycle threshold for each reaction. qPCR Experiments were performed in triplicates. Each qPCR experiment was repeated twice. Gene expression for each gene was normalized to the expression of the housekeeping gene, glyceraldehyde-phosphate-dehydrogenase (*GAPDH*). The relative expression intensity was estimated by calculating 2^−ΔΔCt^ for each sample. For the calculation of the values displayed in Table 2, the control (sham + vehicle) was set as 1. The specificity of all PCR products was checked by a melting curve analysis.

**Table 1:** Primer sequences used for quantitative real-time polymerase chain reaction

Target	Forward Primer 5′–3′	Reverse Primer 3′–5′
m_macrosialin	CTAGCTGGTCTGAGCATCTCT	TTCCACCGCCATGTAGTCC
m_IL1β	ATCACTCATTGTGGCTGTGG	CATCTCGGAGCCTGTAGTGC
m_TNFα	CACAGCCTTCCTCACAGAGC	GGAGGCAACAAGGTAGAGAGG
m_TNFR1	CTGTATGCTGTGGTGGATGG	CCACTACTTCCAGCGTGTCC
m_TNFR2	TCCTCCTGACCTTCTAATGAGC	TCCAACTCACAGTGCCTAACC
m_IL6	GAGGATACCACTCCCAACAGACC	AAGTGCATCATCGTTGTTCATACA
m_gp130	TGAAGCTGTCTTAGCGTGGG	GGTGACCACTGGGCAATATG
m_IL6R	GCTGGCAGCACCCTGAGACC	TCCAAGGAGTGCCCGTGACC
m_MMP9	TGTATGGAGATTCGACTTGAAGTC	TGAGTTCCAGGGCACACCA
m_TIMP1	CCAGAACCGCAGTGAAGAAGT	TCTCCAAGTGCACAAGCCTA
m_COX2	TCATCAGTTTTTCAAGACAGATC	ACCTGATATTTCAATTTTCCATCC
m_iNOS	GAGCAACTACTGCTGGTGGT	CGATGTCATGAGCAAAGGCG
m_IL10	CAGCCGGGAAGACAATAACTG	CCGCAGCTCTAGGAGCATGT
m_GAPDH	TCTCCTGCGACTTCAACA	TGTAGCCGTATTCATTGTCA

**Table 2:** Minocycline regulates the gene expression of various pro-inflammatory and anti-inflammatory cytokines in brain homogenates after SAH

Gene name	SAH + vehicle	SAH + minocycline
macrosialin	2.44 ± 0.1****	1.06 ± 0.2^####^
IL-1β	1.63 ± 0.3***	0.87 ± 0.4^####^
TNF-α	2.51 ± 1.0***	0.91 ± 0.3^####^
TNFR1	0.97 ± 0.2 (ns)	0.81 ± 0.1 (ns)
TNFR2	1.24 ± 0.6 (ns)	0.9 ± 0.2 (ns)
IL-6	2.54 ± 0.6****	0.76 ± 0.5^####^
gp130	0.96 ± 0.2 (ns)	0.94 ± 0.3 (ns)
IL-6R	1.13 ± 0.4 (ns)	1.29 ± 0.3 (ns)
MMP9	1.54 ± 0.24****	1.05 ± 0.1^####^
TIMP1	0.23 ± 0.1**	3.13 ± 2.8^###^
COX2	2.09 ± 1.04****	0.74 ± 0.2^###^
iNOS	3.98 ± 1.27****	0.78 ± 0.5^###^
IL-10	0.38 ± 0.16****	1.57 ± 0.62^####^

Statistical significance was evaluated using one-way analysis of variance (Holm–Sidak method) indicating **^/##^ *P* < 0.01, ***^/###^ *P* < 0.001, and ****^/####^ *P* < 0.0001 versus sham + vehicle (calculated as 1 for each target gene) and SAH + vehicle, respectively. Values are means ± standard error of mean (*n* = 3 animals per group). Each quantitative real-time polymerase chain reaction (qPCR) reaction was performed in triplicates. Each qPCR experiment was repeated twice. Gene expression for each gene was normalized to the expression of the housekeeping gene, glyceraldehyde-phosphate-dehydrogenase (GAPDH). The relative expression intensity was estimated by calculating 2^−ΔΔCt^ for each sample. For the calculation of the values displayed in the table, the control (sham + vehicle) was set as 1. The specificity of all polymerase chain reaction products was checked by a melting curve analysis

IL, interleukin, TNF-α, tumor necrosis factor α, TNFR, tumor necrosis factor receptor, gp130, glycoprotein 130, IL-6R, interleukin-6 receptor, MMP9, matrix metalloproteinase 9, ns, not significant, TIMP1, tissue inhibitor of MMP 1, COX2, cyclooxygenase 2, iNOS, inducible nitric oxide synthase

^*^Significance with respect to sham

^#^Significance with respect to the SAH + vehicle

### Statistical Analysis

Statistical analysis of data was performed through GraphPad Prism 6.1 (GraphPad Software) using analysis of variance with pairwise comparison or the Holm–Sidak method, assuming significance for *P < 0.05, **P < 0.01, ***P < 0.001, and ****P < 0.0001.

## Results

### Minocycline Inhibited Microglia/Macrophage Accumulation due to SAH

To evaluate the effect of SAH on the distribution of microglia/macrophages over a long-term period of 21 days, immunohistochemistry of coronal brain sections with an antibody against Iba1 has been used in vehicle-administered sham versus SAH animals. Iba1 is specifically expressed in microglia and macrophages, both of which are virtually indistinguishable because of the expression of most commonly used cell markers in vivo. However, the specificity of the staining is limited in injured brain tissue, where peripheral macrophages may infiltrate. Cells positive for Iba1 were therefore described as microglia/macrophages throughout the text.

**Fig. 1:**
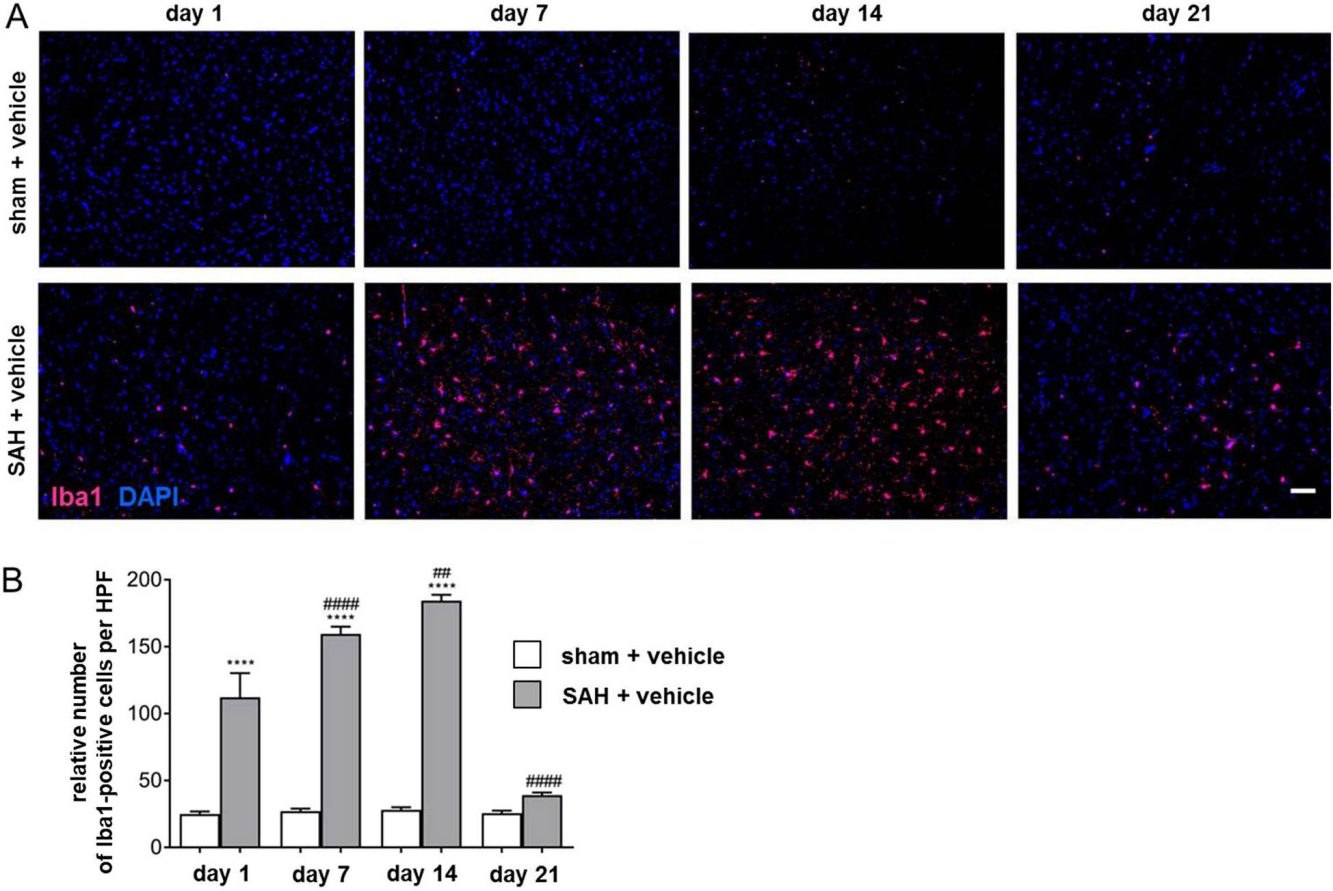
Time-dependent increase of microglia/macrophage accumulation until day 14 after SAH. **a** Representative images of coronal brain sections of sham + vehicle (upper panel) and SAH-operated mice (lower panel) were chosen to demonstrate the time-dependent accumulation of microglia/macrophages in response to the bleeding. Microglia/macrophages were stained using Iba1 (magenta) and counterstained with DAPI (blue) to visualize cell nuclei. The number of Iba1-positive cells in SAH and sham + vehicle (not provided) was counted using ImageJ software. Bar indicates 60 µm. Microglia/macrophage accumulation was significantly increased in SAH + vehicle-treated mice at all experimental time points compared with the respective sham. **b** Microglia/macrophage accumulation culminated 14 days after SAH and was reduced at day 21 after the bleeding when compared with the amount counted at day 14, as depicted. Values from the graph are means ± SEM (*n* = 6 animals per group). ****^/####^*P* < 0.0001, ***^/###^*P* < 0.001, **^/##^*P* < 0.01, and *^/#^*P* < 0.05 versus sham + vehicle and SAH + vehicle, respectively. Statistical significance was determined by one-way ANOVA Bonferroni corrected. (*) describes significance with respect to sham + vehicle; (^#^) describes significance with respect to the earlier experimental time point. ANOVA analysis of variance, DAPI 4',6-diamidino-2-phenylindol, Iba1 ionized calcium-binding adapter molecule 1, SAH subarachnoid hemorrhage, SEM standard error of mean (Color figure online)

A significant time-dependent increase of microglia/macrophage number was observed until day 14 after induction of the bleeding (Fig. 1a, b). On day 21, the amount of Iba1-positive cells was decreased compared with day 14 after SAH. Therefore, for all subsequent experiments going forward, animals were maintained until this experimental time point.

**Fig. 2:**
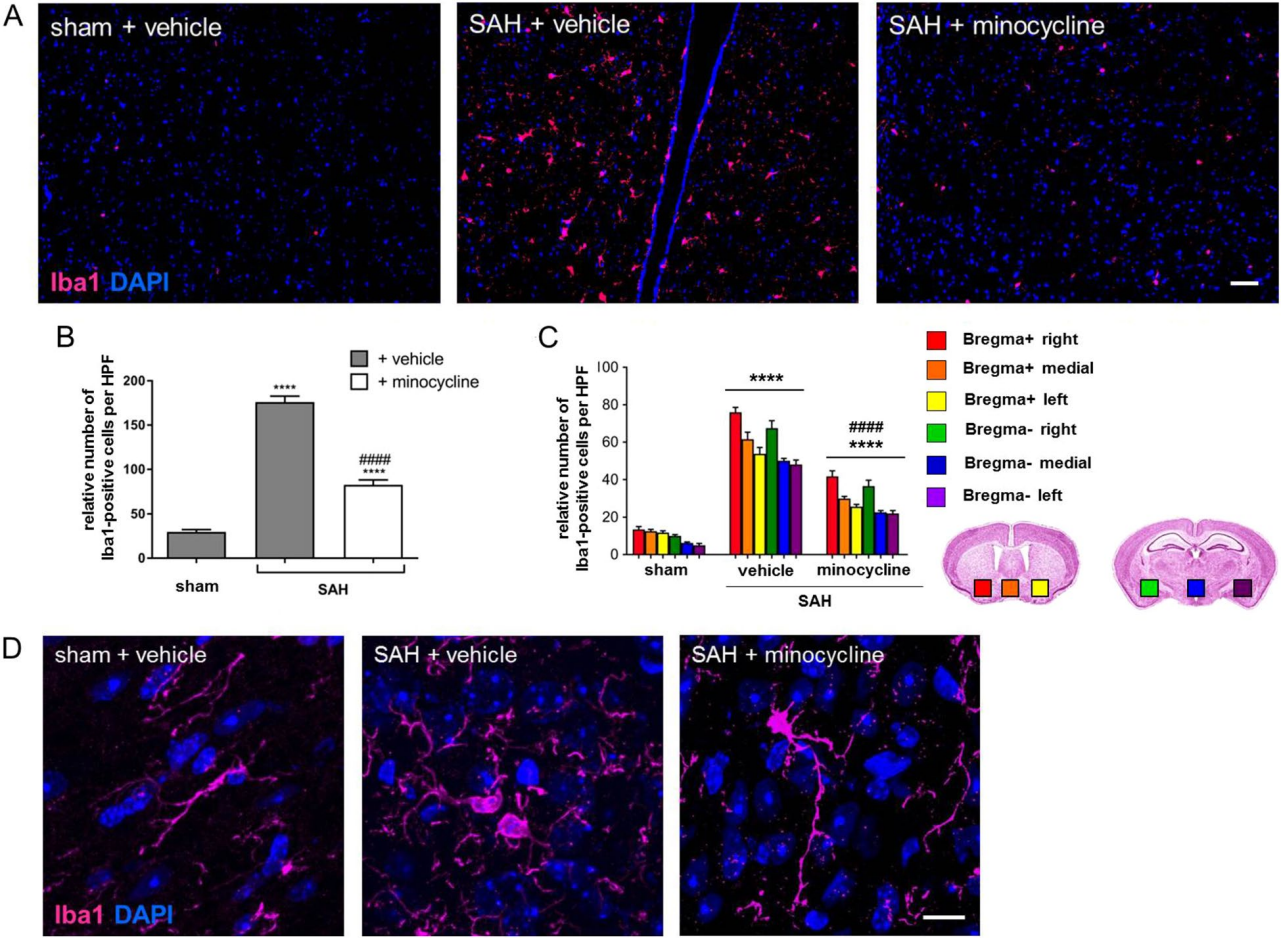
Minocycline-mediated effects on microglia/macrophage accumulation and morphology after SAH. Coronal brain sections of minocycline (45 mg/kg) or vehicle-treated mice were stained for Iba1 (magenta) and with DAPI to visualize cell nuclei. **a** Representative brain sections were processed, and the number of Iba1-positive cells [46] was counted using ImageJ software. Bar indicates 60 µm. Microglia/macrophage accumulation was significantly increased in SAH + vehicle-treated mice compared with sham controls. **b** Minocycline reduced microglia/macrophage accumulation due to SAH. **c** A summary of microglia/macrophage distribution in brain areas analyzed with respect to the puncture, ipsilateral, medial, and contralateral site. Coronal brain slices were analyzed in the area of corpus callosum (bregma + ; the brain with red, orange, and yellow squares) and hippocampus (bregma − ; the brain with green, blue, and purple squares) at the base of the brain, as indicated by colored squares. Values from all graphs are means ± SEM (*n* = 6 animals per group). ****^/####^*P* < 0.0001, ***^/###^*P* < 0.001, **^/##^*P* < 0.01, and *^/#^*P* < 0.05 versus sham + vehicle and SAH + vehicle, respectively. Statistical significance was determined by one-way ANOVA Bonferroni corrected. **d** Representative images indicating microglia/macrophage morphology in response to SAH operation and minocycline administration. Bar indicates 20 µm. Although cell bodies of Iba1-positive cells in sham mice appeared thin and elongated, they were found with the typical amoeboid cell morphology, indicating the activated state in SAH mice. Minocycline administration did not markedly change the cell morphology when compared to sham + vehicle. (*) describes significance with respect to sham; (^#^) describes significance with respect to the SAH + vehicle. ANOVA analysis of variance, DAPI 4',6-diamidino-2-phenylindol, Iba1 ionized calcium-binding adapter molecule 1, SAH subarachnoid hemorrhage, SEM standard error of mean (Color figure online)

Next, the impact of minocycline on the number and activity state of microglia/macrophages in delayed brain injury after SAH was examined. The mean microglia/macrophage density was much higher in the SAH vehicle-treated group then in sham + vehicle controls. A significant increase of Iba1-positive microglia/macrophages was observed in the respective areas (Fig. 2c). Minocycline administration led to a vast reduction of microglia/macrophage density analyzed at the base of the brain (Fig. 2a, b). This minocycline-mediated reduction could be documented in all brain areas analyzed with respect to the puncture (ipsilateral, medial contralateral at the base of the brain parenchyma) (Fig. 2c).

Concerning the activity state of the cells, we observed significant morphological differences between microglia/macrophages found in sham versus SAH-operated vehicle-treated mice (Fig. 2d). Although Iba1-positive cells in sham + vehicle mice were representative for a low stage of activation or an inactive state with thin and elongated cell bodies, there was a large number of cells at higher stages of activation in vehicle-treated SAH mice, as indicated in the representative images in Fig. 2d. Activated microglia/macrophages were characterized by an amoeboid cell body and long processes in vehicle-treated SAH mice. There was a diminished total number of activated amoeboid microglia/macrophages due to minocycline administration post SAH. The morphology of Iba1-positive cells in this experimental group was however characterized by a thick amoeboid cell body and long and relatively thick processes. Therefore, further investigation was needed to assess the impact of the drug on microglia/macrophage activation.

### Minocycline Decreased the Phagocytic Activity of Microglia/Macrophages in Response to SAH

**Fig. 3:**
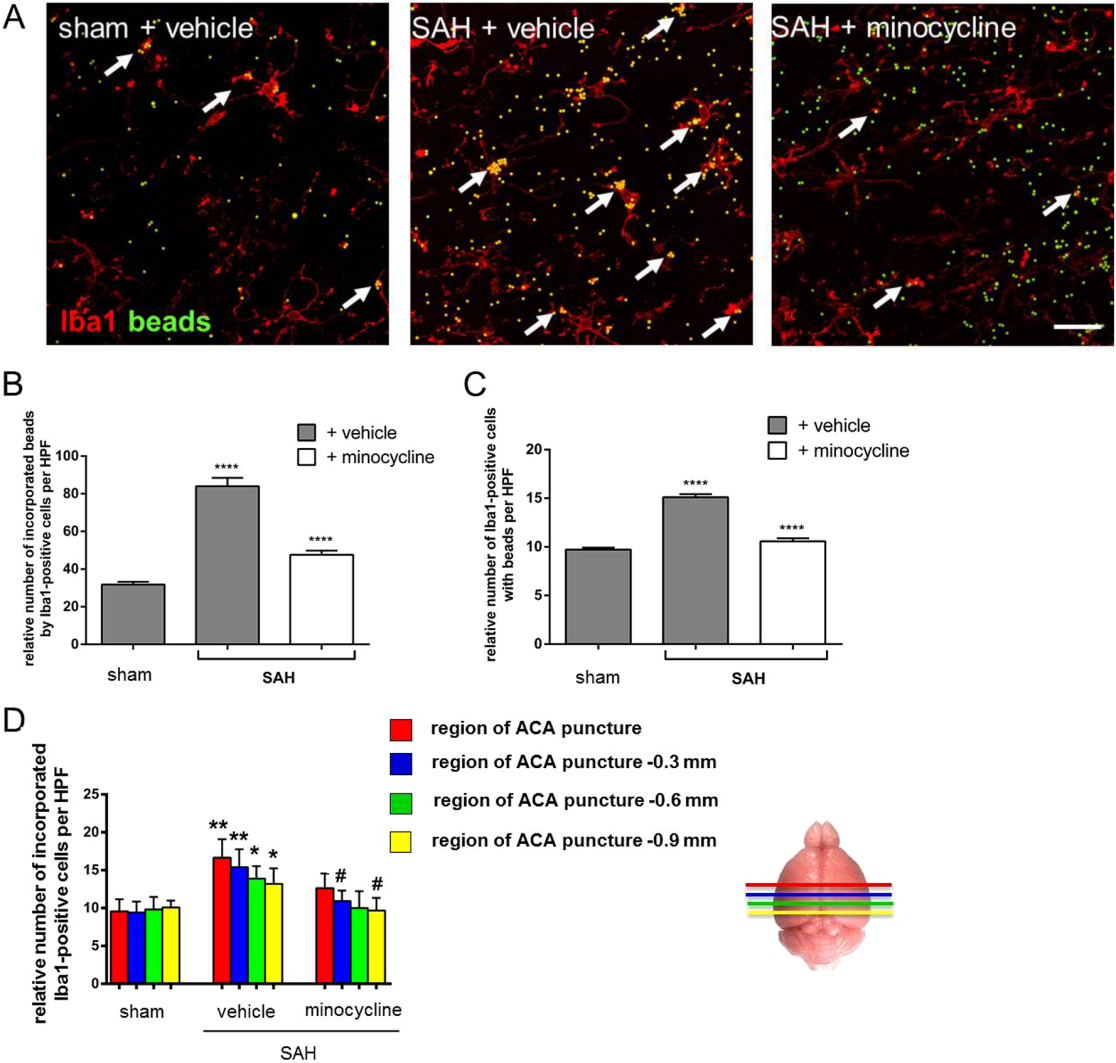
Effects of minocycline on microglia/macrophage phagocytosis post SAH. Mice were operated on day 0 and treated with 45 mg/kg/dose with minocycline or PBS over 14 days. Then acute brain slices were prepared, treated, and stained, and images were recorded, as indicated in the Methods section. **a** Microglia/macrophage phagocytosis was significantly increased because of SAH and reflected by the accumulation of fluorescent beads (green) in Iba1-positive cells [46]. Minocycline significantly reduced phagocytic activity of microglia/macrophages, as depicted by a lower amount of fluorescent beads co-localized with Iba1-positive cells. Representative images were analyzed using ImageJ software. Relative number of fluorescent beads co-localized with Iba1-positive cells (**b**), the relative amount of phagocytic active microglia/macrophages (**c**), and the number of phagocytic active microglia/macrophages assigned to the analyzed brain area relatively to the ACA puncture (**d**) are depicted in the graphs. Bar indicates 20 µm. Values from all graphs are means ± SEM (*n* = 5 animals per group). ****^/####^*P* < 0.0001, ***^/###^*P* < 0.001, **^/##^*P* < 0.01, and *^/#^*P* < 0.05 versus sham + vehicle and SAH + vehicle, respectively. Statistical significance was determined by one-way ANOVA Bonferroni corrected. (*) describes significance with respect to sham; (^#^) describes significance with respect to the SAH + vehicle. ACA anterior cerebral artery, ANOVA analysis of variance, DAPI 4',6-diamidino-2-phenylindol, Iba1 ionized calcium-binding adapter molecule 1, PBS phosphate buffered saline, SAH subarachnoid hemorrhage, SEM standard error of mean (Color figure online)

Phagocytosis plays an essential role in the clearance of CNS tissue from apoptotic cells, extracellular protein aggregates, or infectious particles by microglia/macrophages. It therefore represents an important process maintaining tissue homeostasis in response to distinct molecular signals after an insult-induced inflammation. To evaluate the influence of SAH and minocycline on the phagocytic activity of microglia/macrophages, we used a functional assay on the basis of counting fluorescently labeled beads co-localized with Iba1-positive cells in acute brain slices [24]. Briefly, glass beads were coated with glucose and cross-linked with a lipid bilayer, allowing fluorophore labeling for immunofluorescence analysis. Phagocytosis of microglia/macrophages was induced by SAH, which led to a significant increase in bead incorporation in SAH mice compared with sham + vehicle controls (Fig. 3). The phagocytic activity was strongly attenuated owing to 14 days of minocycline treatment, as reflected by the relative number of incorporated beads by Iba1-positive cells compared with the vehicle-treated animals post SAH. This diminishing effect of minocycline on microglia/macrophage phagocytosis was also depicted by the relative number of microglia/macrophages co-localized with fluorescence-labeled beads in SAH (Fig. 3c, d). Additionally, the relative distribution of phagocytic active microglia/macrophages with beads was estimated by allocating to specific brain regions relatively to the puncture in the three groups, as indicated in Fig. 3e.

In addition to the phagocytic activity that has been assessed by the functional assay, the functional potential and activity of microglia/macrophages after SAH with and without minocycline was further tested by the relative gene expression of macrosialin in whole-brain lysates. A significant induction of macrosialin was found in response to SAH when compared with the sham + vehicle controls, whereas minocycline injection had a clearly lowering effect on its expression (Table 1).

### Attenuation of SAH-Induced Pro-inflammatory Cytokine Expression by Minocycline in Mouse Brain Homogenates

By qPCR, we were able to analyze the influence of SAH + vehicle after minocycline administration in relation to the sham + vehicle controls. As summarized in Table 1, the relative gene expression of several key genes known to be regulated under inflammatory conditions has been evaluated. A robust induction of interleukin-1β (IL-1β), tumor necrosis factor α (TNF-α), and interleukin-6 (IL-6) could be recorded owing to SAH when compared with sham + vehicle. Minocycline led to a downregulation of SAH-induced IL-1β, TNF-α, and IL-6, pointing to an approximate return to the control level. The downregulation of these molecules was significant when related to SAH + vehicle samples. Neither SAH nor minocycline had a meaningful effect on the expression of the IL-6 and TNF-α receptors: IL-6 receptor glycoprotein 130 (gp130) and TNF receptors 1 and 2. Moreover, significant effects of minocycline were observed for the relative gene expression of the endopeptidase MMP9 and its antagonist TIMP1. Minocycline suppressed SAH-induced MMP9 overexpression that reached approximately the level measured in sham + vehicle. The relative expression of TIMP1 was even upregulated by more than threefold in response to minocycline when compared with SAH + vehicle. The effect on the expression of other inflammatory mediators, such as cyclooxygenase 2 and iNOS, was found to be significantly upregulated owing to SAH. Minocycline had a profound lowering effect on SAH-induced overexpression of both molecules. The expression of the anti-inflammatory interleukin-10 (IL-10) was lowered after SAH, whereas minocycline led to a slide overexpression.

### Minocycline Reduced the Spatial Interaction Between Microglia/Macrophages and Neurons Post SAH

**Fig. 4:**
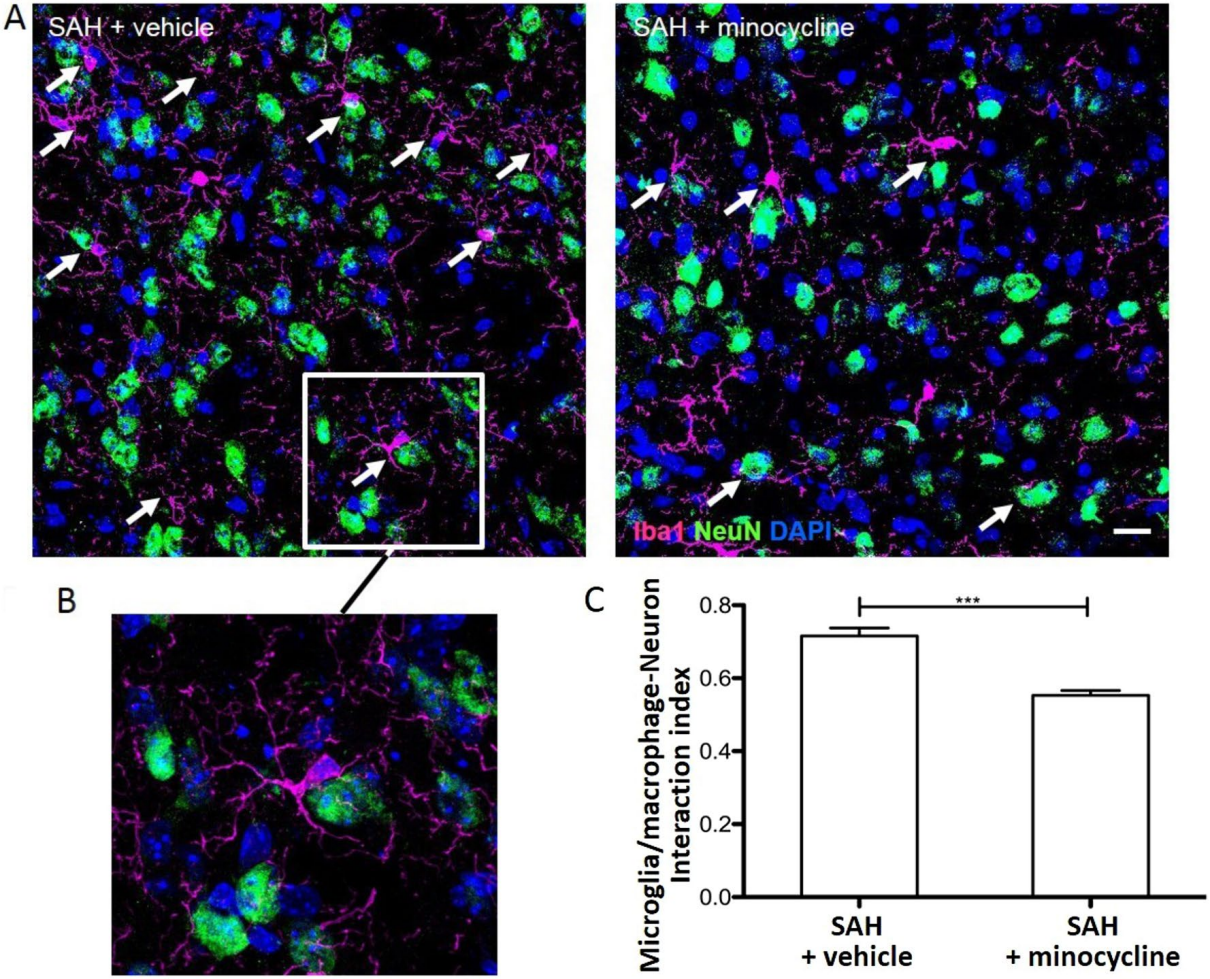
Minocycline attenuated the interaction of activated microglia/macrophages and neurons in SAH-operated mice. Minocycline or vehicle was injected in SAH-operated mice, and brain cryosections were stained for neurons and microglia/macrophages. **a** Representative images of activated Iba1-positive cells (magenta) and NeuN-stained neuronal cell bodies (green) were recorded by confocal microscopy. The spatial interaction of these two cell types was assessed in images by counting the number of Iba1-positive cells contacting or overlapping with NeuN cells by the cell body at least one process. Nuclei were labeled with DAPI. White arrows point to microglia/macrophage interacting with neurons. **b** Magnification of a representative Iba1-positive cell in close vicinity of a neuronal cell. **c** The number of interactive microglia/macrophages related to the number of all activated microglia/macrophages was calculated. Values are means ± SEM (*n* = 6 animals per group, 4 brain slices per animal, and at least 4 images per brain). ***P < 0.001 versus SAH + vehicle. Statistical significance was determined by one-way ANOVA Bonferroni corrected. ANOVA analysis of variance, DAPI 4',6-diamidino-2-phenylindol, Iba1 ionized calcium-binding adapter molecule 1, SAH subarachnoid hemorrhage, SEM standard error of mean (Color figure online)

Microglia/macrophages are well known to interact with neurons and regulate several aspects of neuronal functions. The spatial interaction between these two cell types was analyzed by counting activated Iba1-positive cells physically interacting with NeuN immune-labeled cells in coronal brain slices obtained from SAH-operated mice. We found a more prominent microglia/macrophage–neuronal apposition either with the cell body or with more than one process in SAH-operated vehicle-treated mice (Fig. 4a). The interaction of microglia/macrophages with neurons has been specified by an index relating the amount of microglia/macrophages co-localized and approximated to at least one neuronal cell to the number of all activated Iba1-positive microglia/macrophages counted per HPF (Fig. 4b). Microglia/macrophage–neuronal interaction was significantly reduced by minocycline administration post SAH, as reflected by the representative images and the interaction index provided in Fig. 4c.

### Minocycline Prevented SAH-Induced Neuronal Cell Death 14 days after Induction of the Bleeding

**Fig. 5:**
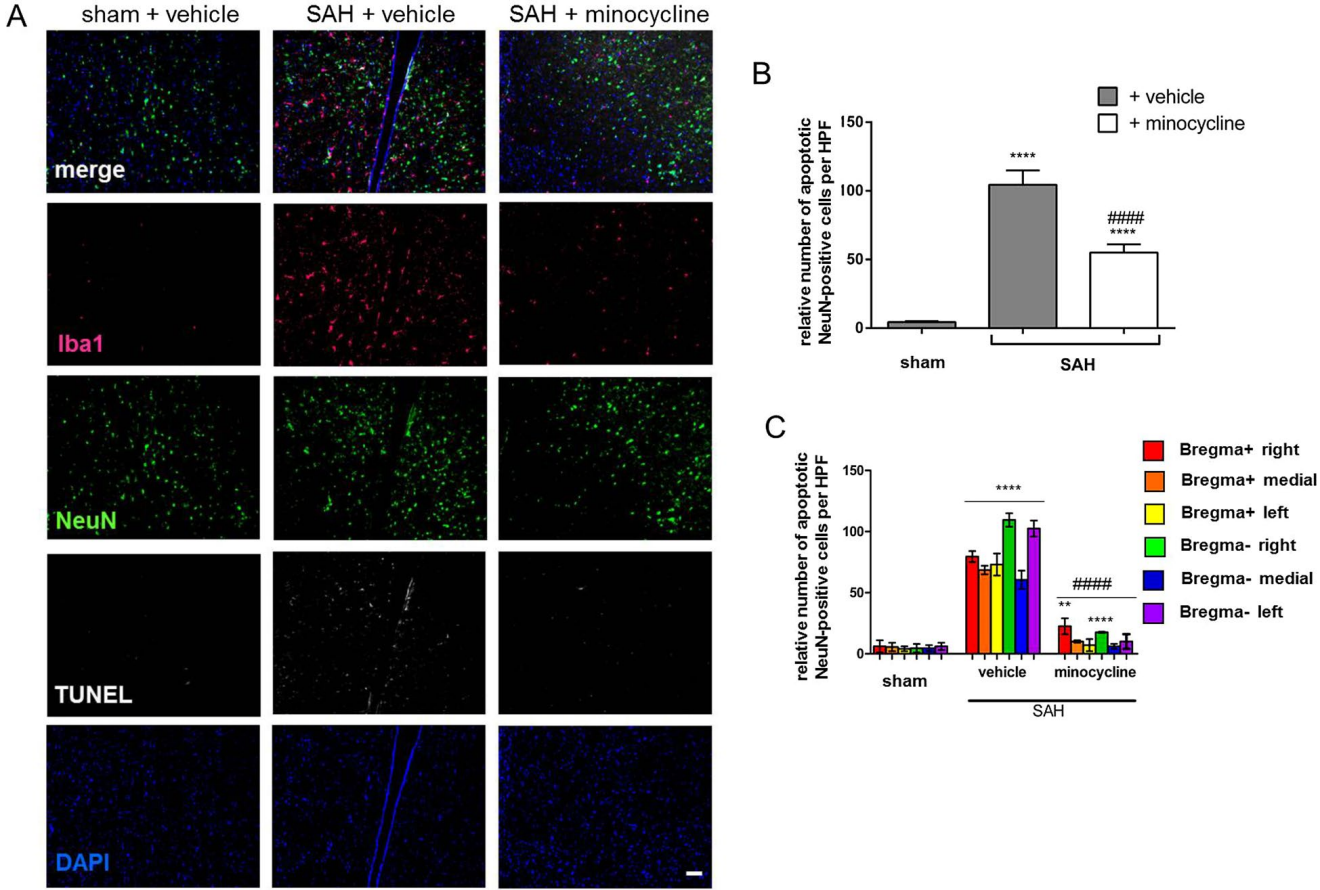
SAH-induced neuronal cell death was significantly lowered on minocycline administration. **a** Mice were operated and treated as indicated. **b** TUNEL-positive (white) NeuN-labeled and DAPI-labeled (blue) neuronal cell bodies (green) were counted, and the results were summarized. Microglia/macrophages were stained by Iba1. **c** Regional distribution of TUNEL-NeuN-DAPI double-triple-positive neurons is depicted. Coronal brain sections were analyzed in the area of corpus callosum (bregma +) and hippocampus (bregma −), as indicated by colored squares in Fig. 2c. Bar indicates 20 µm. Values from all graphs are means ± SEM (*n* = 6 animals per group). ****^/####^*P* < 0.0001 versus sham + vehicle and SAH + vehicle, respectively. Statistical significance was determined by one-way ANOVA Bonferroni corrected. (*) describes significance with respect to sham; (^#^) describes significance with respect to the SAH + vehicle. ANOVA analysis of variance, DAPI 4',6-diamidino-2-phenylindol, Iba1 ionized calcium-binding adapter molecule 1, SAH subarachnoid hemorrhage, SEM standard error of mean, TUNEL terminal deoxynucleotidyl transferase deoxyuridine triphosphate nick end labeling (Color figure online)

To further address whether SAH-induced neuronal cell death could be prevented by minocycline, TUNEL-positivity in NeuN cells was assessed in the three experimental groups. Compared with sham + vehicle animals, we found a significantly increased number of TUNEL-NeuN-DAPI-positive cells in SAH mice (Fig. 5). In contrast, a significantly reduced neuronal cell death was documented in response to minocycline administration. Accordingly, the number of vital neurons was significantly higher in minocycline-treated animals, as reflected by the representative immunofluorescent staining and as indicated in Fig. 5a, b. The distribution of neuronal cell loss at the base of the brain is depicted in Fig. 5c.

## Discussion

The potential role of minocycline as a wide-range anti-inflammatory agent for the treatment of diverse inflammatory diseases of the CNS, including delayed brain injury post SAH, has stimulated diverse research interests [21–23]. Besides mechanistic insights for the pharmacological action of minocycline at early time points after SAH described by others, Sherchan et al. [22] provided histological data describing long-term benefits of minocycline administration post SAH in a comprehensive neurophysiological study. A detailed description of minocycline’s impact on microglia/macrophage function and its influence on neuronal survival and the inflammatory response due to delayed brain injury post SAH required, however, further investigation.

The principle novel finding of our current study confirms the observations of others [22] that the promising multifunctional drug minocycline provides a robust long-term improvement to the inflammatory response due to secondary brain injury after experimental SAH in mice. To our knowledge we are the first to show that the phagocytic activity of highly accumulated microglia/macrophages is significantly reduced by minocycline treatment post SAH, as indicated in Fig. 3. This finding coincides with a lowered number of apoptotic neurons (Fig. 5) and reactive astrocytes (Supplementary Fig. S2). We further demonstrate that the expression of important inflammatory markers was significantly regulated by the drug after SAH, underlining its clear anti-inflammatory properties (Table 1). On the basis of our data and the results of others, we speculate that the beneficial effect minocycline on SAH-induced neuronal cell loss may be attributed to the direct influence of the drug on microglia/macrophage-mediated inflammation.

Mechanisms leading to secondary brain injury remain insufficiently understood. As it was described in human and rodent studies, the brain’s innate microglia along with macrophages infiltrating from the periphery are attracted to the site of the injury and accumulate around the affected areas. In line with findings, i.e., in neurodegenerative diseases such as Alzheimer disease, in which sustained innate immune activation is associated with amyloid plaques, we were able to identify a massive increase of Iba1-positive microglia/macrophages over time culminating on day 14 after experimental SAH. This phenomenon has been extensively studied by using a transgenic microglia-depleted mouse model (HSVTK), and we could convincingly demonstrate that these accumulating Iba1-positive cells originated from resident microglia [8, 25, 26]. We observed that microglia/macrophages first accumulate at the site of the injury, followed by a spreading distribution over other brain regions, thereby supporting and exacerbating the SAH-induced brain damage. Although the pro- and anti-inflammatory state of activated microglia/macrophages is a topic of debate, short-term activated microglia/macrophages were considered beneficial, whereas their prolonged activation mediates damaging responses to injury [27, 28]. Chronically activated microglia/macrophages constitute the main source of pro-inflammatory cytokines and proteases in the inflamed brain [29]. In accordance with these facts, the massive accumulation of microglia/macrophages significantly contributes to the progressing inflammatory response due to secondary brain injury post SAH. We have shown here that the number of Iba1-positive microglia/macrophages in the surveillance state characterized by the typical activated cell morphology was increased in coronal brain sections of SAH-operated mice. As compared with thin and elongated microglia/macrophages present in the controls, activated microglia/macrophages appeared with an amoeboid cell body and long processes in SAH specimens (Fig. 2). Additionally to this morphological comparison, a raised microglia/macrophage activity was further confirmed by functional assays addressing the ability of Iba1-positive cells to incorporate fluorescently labeled beads via phagocytosis (Fig. 3). Supportive evidence for the phagocytic capability of microglia/macrophages in SAH-operated mice comes from the overexpression of macrosialin (Table 1). An overexpression of macrosialin being the murine homologue of CD68 indicates a phagocytically active state of cells, including microglia/macrophages, which digest internalized material due to accumulated lysosomal vacuoles [30]. Minocycline was reported to be generally used as a selective microglial/macrophage inhibitor [31]. Moreover, the dose used in our present study, 45 mg/kg and, shown to produce a reducing effect on the accumulation of microglia/microphages in the injured brain after SAH (Fig. 2). This dose also lowered the expression of macrosialin (Table 1) and attenuated the microglia/macrophage–neuronal interaction in the context of SAH (Fig. 4). Despite these numerous indications for the microglia/macrophage-deactivating ability of the drug, we were not able to define significant morphological differences between the SAH-vehicle and minocycline-treated microglia/macrophages (Fig. 2). Considering the roundish cell body and thickened processes, Iba1-positive cells appeared activated in the SAH + minocycline group.

Independent of its anti-microbial and iron-chelating action, the mechanism of minocycline involves multiple sites, including iNOS, prostaglandin E2, free radical formation, the downregulation of pro-inflammatory cytokines such as TNF-α, IL-1β, and IL-6, and an upregulation of the anti-inflammatory cytokines IL-10, as demonstrated here by the analysis of the expression of these molecules in whole-brain lysates (Table 1) [32–34]. IL-10 is a pleiotropic immunoregulatory cytokine with cytoprotective effects having a crucial role in the development of the anti-inflammatory milieu associated with tissue repair, i.e., after stroke [35]. Minocycline is moreover known to exert anti-inflammatory effects by inhibiting matrix metalloproteases, cyclooxygenase 2, and phospholipase A2 [36]. Although we did not specify the respective cell types being responsible for the overexpression of the herein analyzed pro-inflammatory markers, we reveal a significant anti-inflammatory action of the drug by lowering the expression of several cytokines involved in the regulation of apoptotic pathways and immunomodulation after SAH.

Besides activated microglia/macrophages, reactive astroglia, the number of which was significantly increased after SAH (Supplementary Fig. S2), might also contribute to the development of cerebral inflammation [37]. In the course of our experiments, we also observed a minocycline-mediated reduction of GFAP-positive reactive astroglia after SAH on day 14 (Supplementary Fig. S2). In this line, an augmented synthesis of pro-inflammatory mediators by glia may cause a positive feedback loop in paracrine release of cytokines by other cell types, including neurons, forming a vicious cycle of sustained inflammation [38]. This phenomenon was well documented, i.e., for TNF-α, in which the binding to its receptor on the same or a neighboring cell can initiate a cascade of signal transductions via activating several transcription factors, including nuclear factor κB [39]. This in turn may lead to the production of additional pro-inflammatory cytokines ultimately impacting neuronal survival [38, 40].

On the basis of our previous findings showing the loss of neuronal density, which was attributed to the activation of resident microglia rather than by infiltrating macrophages after SAH, we have further hypothesized modulatory effects of minocycline on neuronal cell death as a consequence of the inhibition of the innate immune system. Applying immunohistochemistry, we could demonstrate a significantly diminishing effect of the drug on the amount of TUNEL-positive neurons due to SAH appearing concomitantly to the decrease of microglia/macrophage accumulation (Fig. 5). In this regard, we have also observed that SAH induced a close vicinity of microglia/macrophages and neurons (Fig. 4). This close interaction has been described to be crucial for the induction of neuronal cell death [41]. We could find here that Iba1-positive microglia/macrophages in the vicinity of neurons were in an activated state and the number of microglia/macrophages interacting with NeuN-positive neurons could be robustly decreased by means of minocycline treatment. Tikka et al. [42] could already demonstrate that by inhibiting the p38 mitogen-activated protein kinase, minocycline acts as a microglia/macrophage antagonist without specific effects on neuronal function and activity. Taking this fact and our data into consideration, we speculated that the neuroprotective effects of minocycline that could be documented here and were reported by others in the context of SAH may therefore rather indirectly result from the reduction of activated microglia/macrophages [21–23]. However, the association between the microglial/macrophage-deactivating properties and the attendant reduction of neuronal cell death in the herein observed time window of 14 days after SAH is not clear-cut. Because we do not provide detailed mechanistic evidence substantiating, this issue still awaits a specific elucidation. The absence of evident mechanistic proofs of this concept may be therefore considered as a major shortcoming of the herein presented study. In addition, it should be mentioned that the modulatory effect of minocycline on neuronal cell loss along with the downregulation of pro-inflammatory mediators may primarily result from the iron-chelating properties of the drug and secondarily come from the inhibition of glia. Iron-chelating properties of minocycline and other iron chelators need urgently to be taken into account in studies of this sort because oxidative injury due to hemoglobin and iron overload is known to be a major contributor to neuronal damage following all forms of cerebral hemorrhage [43].

Neurological, neurocognitive, or neurobehavioral testing describing the outcome of mice included in the experiments would have been supportive to proof if structural changes of the brain tissue correlated with a worsening of the clinical outcome of the animals. In rats, extensive neurological and mental testing has been established and serves as a good read-out parameter for clinical testing. Although this testing is commonly applied in the SAH perforation model, extensive neurological and neurobehavioral testing in mice suffering from SAH in this certain experimental setup did not reveal any measurable changes between the groups (data not published). One hypothesis causing this dilemma might be the relatively small subarachnoid spaces in mice, resulting in lesser blood volumes in single-perforation models. Using a double-hemorrhage model, most of the animals die, whereas the rest is severely disabled, also hinting at a more narrow threshold of bleeding that is survivable by the much smaller animal. Like in humans, only subtle neurocognitive decline might follow milder hemorrhage patterns. Using the existing testing battery, these might be easily overseen in animals. However, the authors agree that a reasonable assessment of the neurological score should be addressed in the future to show whether structural changes do indeed result in a clinically relevant problem.

Another shortcoming of our study is the dose of the drug (45 mg/kg) used for intraperitoneal injections in our experiments being not congruent with the dose in clinical use. However, several works reporting minocycline administered intraperitoneally in the herein applied concentration to act as a strong anti-inflammatory agent in cerebrovascular diseases, such as SAH and stroke (e.g., Vellimana et al. [44]), described that 45 mg/kg/dose of minocycline strongly reduces neuroinflammation in experimental SAH induced by the endovascular filament perforation in mice. Li and McCullough [45] have given evidence that this particular dose has neuroprotective effects following experimental stroke in mice. A dose–response or a maximum tolerable dose study paired with a test if minocycline alone might cause microglia/macrophage activation under normal conditions would however bring further valuable insights into the mechanistic proof of the drug under normal condition.

## Conclusions

Summarizing, we are the first to demonstrate the potential of minocycline to reduce the number and phagocytic activity of microglia/macrophages in a long-term time window of 14 days after SAH. This was concomitant to a significant reduction of neuronal cell death and a lower number of reactive astrocytes. We further showed a profound impact of minocycline on the expression profile of differential pro- and anti-inflammatory markers. The herein presented findings contribute to the understanding of the long-term benefits resulting from minocycline treatment after secondary brain injury due to SAH.

## Supplementary Information

Below is the link to the electronic supplementary material.

Supplementary file1 (DOCX 47 kb)

Supplementary file2 (DOCX 153 kb)

## Abbreviations

ACA: Anterior Cerebral Artery
ANOVA: Analysis of Variance
ACSF: Artificial Cerebrospinal Fluid
BBB: Blood–Brain Barrier
CNS: Central Nervous System
COX2: Cyclooxygenase 2
CytC: Cytochrome C
DAPI: 4',6-Diamidino-2-Phenylindol
gp130: Glycoprotein 130
HPF: High Power Field
iNOS: Inducible Nitric Oxide Synthase
Iba1: Ionized Calcium-Binding Adaptor Molecule-1
IL: Interleukin
MMPs: Matrix Metalloproteinases
MAPK: Mitogen-Activated Protein Kinase
NFκB: Nuclear Factor Kappa-B
PBS: Phosphate Buffered Saline
PI3K: Phosphoinositide 3-Kinase
SAH: Subarachnoid Hemorrhage
SEM: Standard Error of Mean
TIMP1: Tissue Inhibitor of Metalloproteinase
TUNEL: Terminal Deoxyuridine Triphosphate-Nickend Labeling
TNF: Tumour Necrosis Factor
