# Supplementary material for: Minocycline Attenuates Microglia/Macrophage Phagocytic Activity and Inhibits SAH-Induced Neuronal Cell Death and Inflammation
Source: Neurocrit Care. 2022 May 18;37(2):410–23. doi: 10.1007/s12028-022-01511-5 (PMC9519684; doi:10.1007/s12028-022-01511-5)
Supplement: Supplementary file 1 — Supplementary file1 (DOCX 47 kb) [file 12028_2022_1511_MOESM1_ESM.docx]

**Supplemental Figure S1. Kaplan-Meier curve displaying post-hemorrhagic mortality during a 14 day observation period in the three experimental groups.** No mortality was documented in the sham +vehicle group consisting of 26 animals. Within the SAH +vehicle-treated group (26 mice), 2 animals died within 4 hours (7.69%), 2 died 12 hours after the onset (7.69%), 1 died after 24 hours (3.85%) and 1 mice out of 26 (3.85%) died 48 hours post SAH induction. Within the minocycline-administered group, out of 18 animals, 2 died 12 hours (11.11%) and 1 24 hours after the operation (5.55%). There was no mortality between day 2 and 14 after induction of the bleeding in our study. A total mortality rate of 23.08% was calculated in the SAH +vehicle-treated group. The minocycline-administered group exhibited a total mortality rate of 16.67%.

**
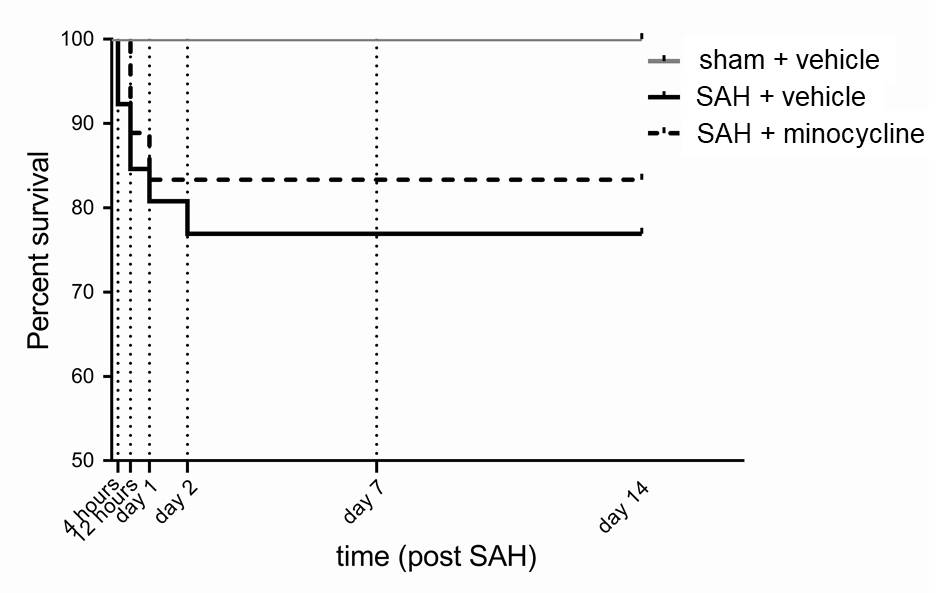
**
