# Supplementary material for: Minocycline Attenuates Microglia/Macrophage Phagocytic Activity and Inhibits SAH-Induced Neuronal Cell Death and Inflammation
Source: Neurocrit Care. 2022 May 18;37(2):410–23. doi: 10.1007/s12028-022-01511-5 (PMC9519684; doi:10.1007/s12028-022-01511-5)

**Supplemental Figure S2. Minocycline blocks SAH-induced GFAP-positive astroglia accumulation.** Coronal brain slices from the three experimental groups were stained for glial fibrillary acidic protein (GFAP, yellow) and DAPI and examined by fluorescent microscopy. **A**: Representative images from each experimental group. GFAP-positive cells were counted and the results were summarized in the graph (**B**). A strong activation of astroglia could be detected in the SAH+vehicle-treated group when compared with sham +vehicle, as reflected by the approximately 4.5-fold increase of GFAP-positive cells. To verify the effect of minocycline on the number and distribution of reactive astroglia, the positivity for GFAP in the three groups was related to the sham as well as to the SAH group. The hemorrhage-induced increased distribution of reactive astroglia was largely blocked by minocycline, which decreased to approximately 2-fold of the sham +vehicle control. Minocycline administration, however, did not reduce the amount of reactive astrocytes to the sham +vehicle control level. Bar: 20 µm. Values from all graphs are means ± SEM, (n=6 animals per group), ****/^####^*P*<0.0001, **/^##^*P*<0.01 versus sham +vehicle and SAH+vehicle, respectively, statistical significance determined by One-Way ANOVA Bonferroni-corrected.


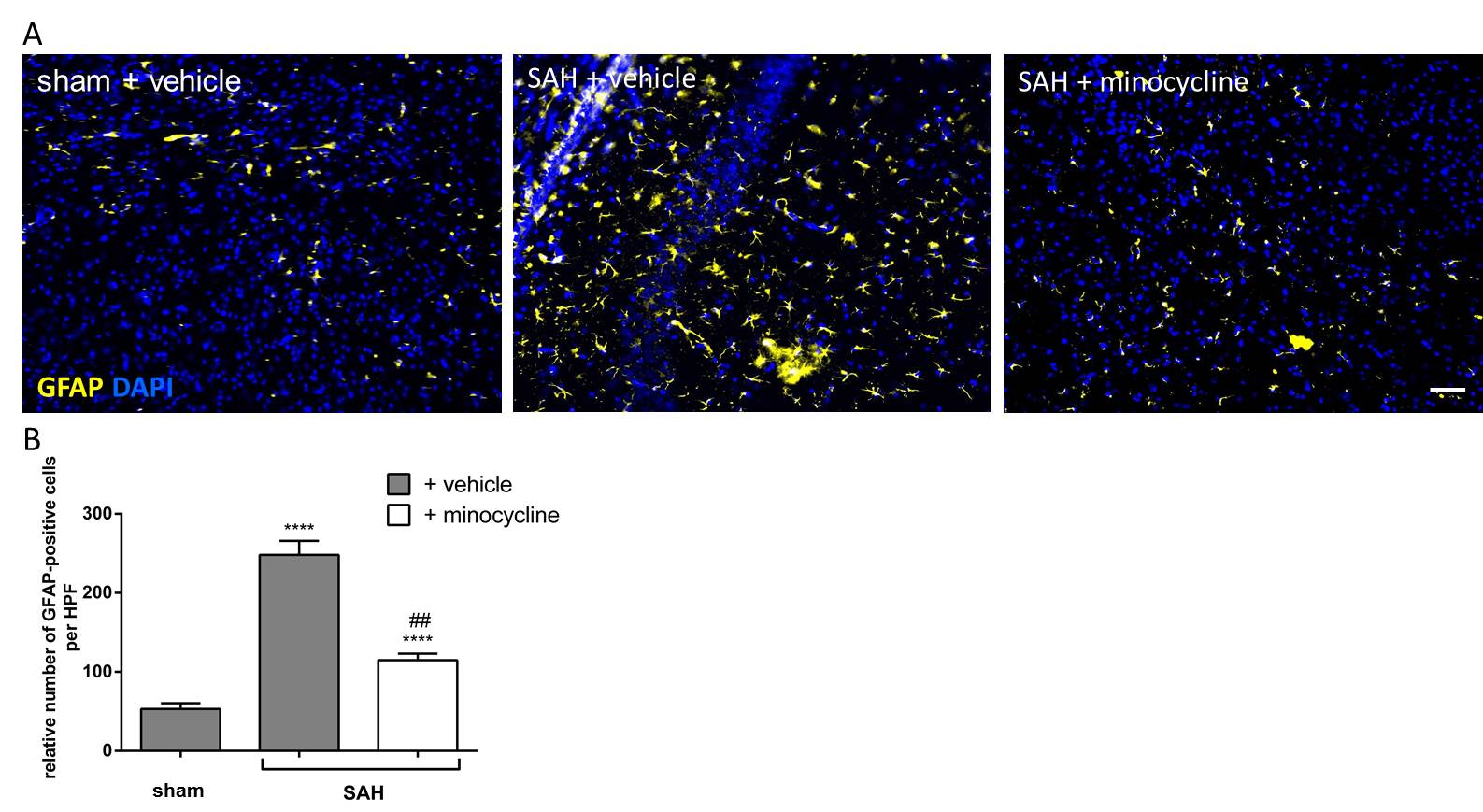

Supplement: Supplementary file 2 — Supplementary file2 (DOCX 153 kb) [file 12028_2022_1511_MOESM2_ESM.docx]
